# Human socio-technical evolution through the lens of an abstracted-wheel experiment: A critical look at a micro-society laboratory study

**DOI:** 10.1371/journal.pone.0310503

**Published:** 2024-11-11

**Authors:** Anders Högberg, Marlize Lombard, Albin Högberg, Eva Iliefski-Janols, Gustaf Lindblad, Alexander Almér, William Hedley Thompson, Mattias Rost, Sebastian Andreasson, Alexander Wiig, Peter Gärdenfors

**Affiliations:** 1 Archaeology, Linnaeus University, Kalmar, Sweden; 2 Palaeo-Research Institute, University of Johannesburg, Auckland Park, South Africa; 3 Department of Applied IT, Gothenburg University, Gothenburg, Sweden; 4 Department of Clinical Neuroscience, Karolinska Institutet, Stockholm, Sweden; 5 Cognitive Science Department of Philosophy, Lund University, Lund, Sweden; New York University, UNITED STATES OF AMERICA

## Abstract

Micro-society experimental setups are increasingly used to infer aspects of human behavioural evolution. A key part of human society today is our dependence on, and use of, technology–whether simple (such as a knife) or complex (such as the technology that underpins AI). Previously, two groups of researchers used an abstracted-wheel experiment to explore the evolution of human technical behaviour, reaching fundamentally different outcomes. Whereas one group saw their results as indicating social learning only (void of causal understanding), the other inferred non-social technical reasoning as part of human technical behaviour. Here we report on the third generation of the micro-society abstracted-wheel experiment. We argue that causal reasoning is inseparable from both social learning and technical reasoning, and that these traits probably co-evolved into the current human socio-technical niche. Based on our outcomes, we present a critical assessment of what this experiment may (or may not) reveal about the evolution of human technical behaviour. We show that the abstracted-wheel experiment reflects behavioural output only, instead of testing for cognition. It is therefore limited in its ability to inform on aspects of human cognitive evolution, but it can provide useful insights into the interrelatedness of social learning, technical reasoning, and causal reasoning. Such a co-evolutionary insight has the potential to inform on aspects of human socio-technical evolution throughout the Pleistocene.

## Introduction

Humans exist in a unique socio-technical niche. We cannot survive without other humans, nor without complex technologies that provide food, shelter, and warmth. The cumulative evolution of this niche is much researched, yet explained differently. Some argue that complex technologies developed through generational social learning or non-social technical reasoning–without involvement of explicit causal understanding [1–3]. Others maintain that non-social technical reasoning is key to the understanding of technical systems and how to improve upon them [4–7], and that causal cognition is necessary for cumulative technological development [7–11].

**Fig 1:**
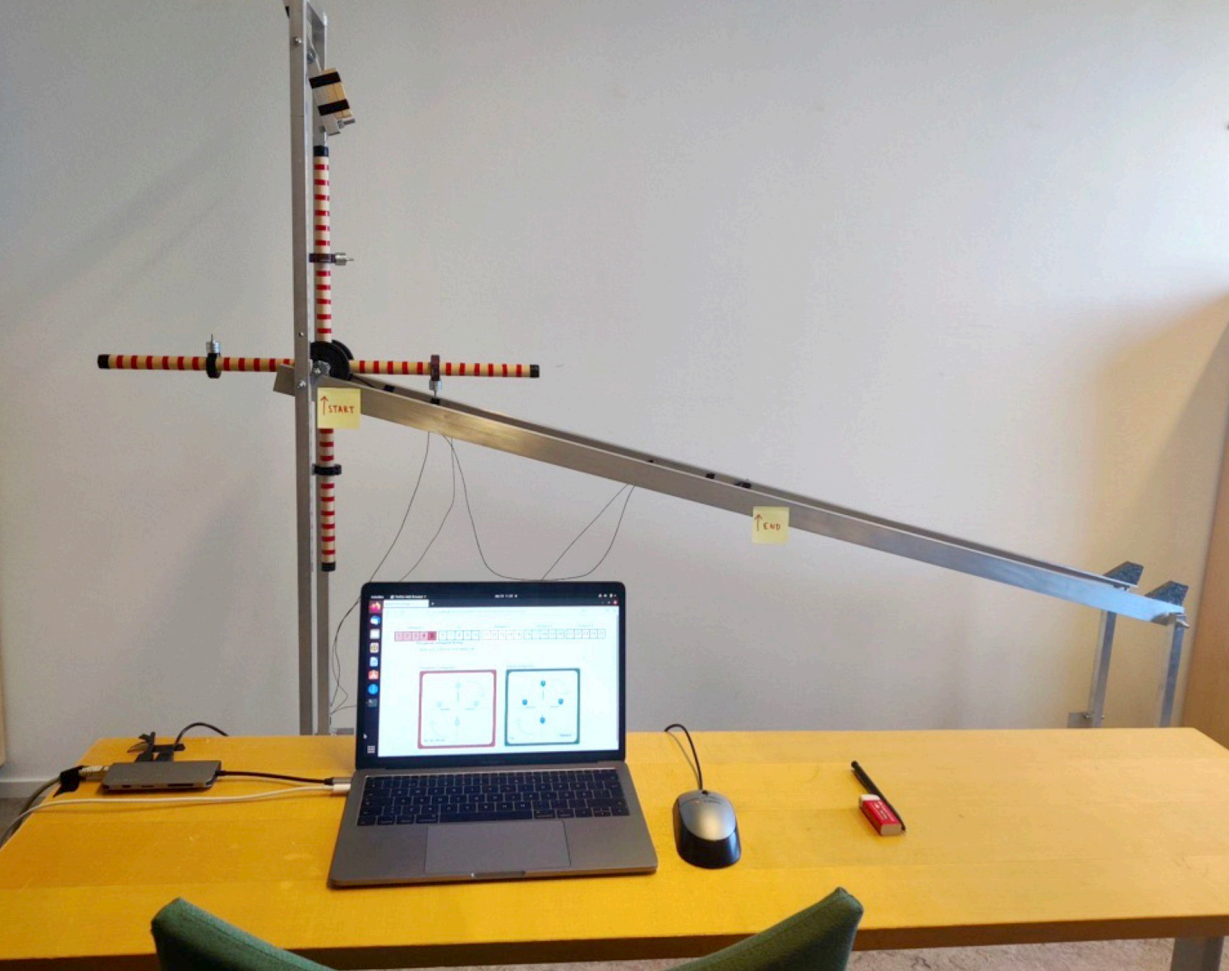
The set up for the abstracted-wheel experiment. The wheel is to roll down the inclined track from the position displayed in the figure. The notes on the track mark the distance, one metre, relevant for time tracking. Participants do not interact with the physical wheel. Instead, they chose their desired weight configurations through the displayed interface on the computer. Photo by Albin Högberg, slightly edited.

The different positions have recently been explored through different versions of a micro-society abstracted-wheel experiment [3, 6] (Fig 1). The experiment is based on a method defined as “linear transmission chains” ([12], C6P6). Participants are given the task to optimise the operation of an abstracted wheel. This is done by altering the weight distribution on the wheel’s four spokes to manipulate its performance and to minimise the time it takes to travel along an inclined track. Participants are organised into chains of five, where each chain position and participant represent a separate artificial generation in a generational transfer chain. The performance and results of each participant are passed on to the next participant in the chain, whose output in turn is given to the next participant. By testing variations in how knowledge is transferred between participants, the purpose is to explore how “a physical artefact becomes progressively optimized across generations” ([3], page 446). To increase the number of independent observations, multiple linear chains are explored [12].

The abstracted-wheel experiment was first presented by Derex et al. [3] (here: ‘the Lille experiment’). They proposed that their results show that the early development and use of technologies such as the bow-and-arrow, shelter building and watercraft emerged from iterative improvements across generations. Hence, they [3] see a basic form of social learning–not causal cognition–as the driver of Pleistocene technological evolution. The second version of the experiment [6] (here: ‘the Lyon experiment’) is a partial replication of the Lille experiment. From their results, Osiurak et al. [6] highlight the importance of non-social technical reasoning in the understanding and refining of technical systems. While recognising the role of social learning, they [6] suggest it isn’t the sole driver of our technical expertise.

As discussed by Mesoudi [12], micro-society laboratory experiments–such as the abstracted-wheel experiment–have become increasingly important in research on cultural evolution. Also, results from the first version of the abstracted-wheel experiment [3] has gained wide-ranging cross-disciplinary attention outside evolutionary studies [13, 14], with influence in, for example, educational science [15], psychology [16], game-theory, social behaviour and learning [17, 18], computer science and algorithmic-human hybrid problem solving [19], psychological processes involved in tool use [20], neurocognitive foundations of technical reasoning [21], and research on normative conflict, change and group-selective forces [22].

Because the Lille [3] and Lyon [6] versions of the abstracted-wheel experiment yielded contradicting results, and in response to the wide-ranging impact of the Lille experiment, we suggest that it is important to critically engage with the relevance of the abstracted-wheel experiment in terms of human socio-technical evolution. Our approach is to partly replicate the experiment and to generate new data to work with. To maintain continuity, we follow the definitions presented in the previous versions of the experiment [3, 6], while simultaneously expanding the experimental set-up.

First, we report on findings based on our version of the abstracted-wheel experiment (here: ‘the Gothenburg experiment’). Then, building on results from all three variants of the experiment, i.e. the Lille, Lyon, and Gothenburg experiments, we explore what they possibly reveal about social learning, technical reasoning, and causal reasoning. We show that a co-evolutionary position, weaving together various aspects of social learning, technical reasoning, and causal reasoning, offers the most reasonable explanation for the human socio-technical niche. We conclude that social learning is inseparable from causal reasoning, technical reasoning is affected by social transmission, and causal reasoning is a combination of social and technical reasoning. To understand these results in a wider context, we further discuss possible confounding aspects of this micro-society experiment as a model for human Pleistocene socio-technical evolution. Note, although we are aware of the concepts being intertwined (see e.g., discussion in Lemonnier [23]), when referring to ‘social learning’ and ‘technical reasoning’, we do so based on how these concepts are presented in the two previous versions of the abstracted-wheel experiment. Derex et al. [3] explored social learning in the absence of causal reasoning. This is in line with what Haidle ([24], page 202) discussed as unidirectional learning where “learner A includes the performance of model B into his/her learning environment”. Consequently, a more elaborate understanding of social learning that also includes aspects of causal understanding, as for example presented by Nielsen et al. [25], is not included. Along similar lines, we draw on the definition of technical reasoning presented by Osiurak et al. [6], a definition that infers non-social technical reasoning as part of human non-social technical behaviour (Table 1).

**Table 1:** Social learning and technical reasoning as elaborated on in the previous versions of the experiment, and causal reasoning as we use it here.

**Social learning**	The process of social interaction between actors in a network producing an accumulation of socio-technical improvements, which goes beyond the individual and becomes situated within the wider social context ([3], page 446).
**Technical reasoning**	An individual ability to learn by non-social cognitive skills situated in the technological domain. The ability to reason implicitly (non-verbally) about physical object properties, transferring what is learnt from one situation to another ([6], page 1643).
**Causal reasoning**	The understanding of cause-effect relations observed in physical, social, and imaginary (predictive) dimensions and how they relate to, and impact upon, each other [9, 11].

## Materials and methods

### The Gothenburg experiment

**Fig 2:**
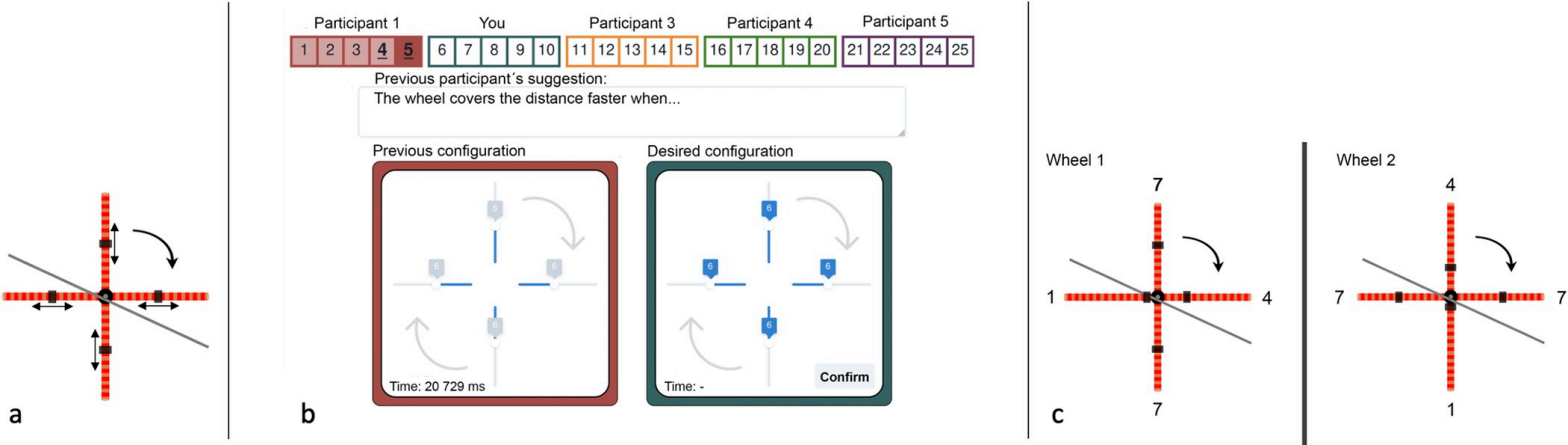
Experimental task and design. (**a)** Illustration of the abstracted-wheel system. The movable weights were presented to participants in position six on each spoke (see also Fig 1). (**b**) Computer interface. Following Derex et al. [3], participants did not interact with the physical wheel but with an abstracted-wheel system using a computer interface. Each participant had five trials (1–5; 6–10; etcetera) to optimise the speed of the wheel. They performed the task as one in a chain of five participants (five generations). While participants in our first and second conditions received (except the first participant in each chain) and transferred (except the last participant in each chain) the last two configurations with wheel speed of each participant (marked with underline and bold for Participant 1, and exemplified as “Previous configuration”), participants in the third condition (Selective learning group) received and transferred the two best configurations. (**c**) After the five trials, the participants completed a knowledge test. For each participant, ten wheel pairs were presented separately (here exemplified with one wheel pair). Participants were asked to determine which of the two wheel-configurations would be the fastest in the experiment they had just completed (wheel 2 in the pair exemplified here) (see also S1 File).

In the Gothenburg version of the abstracted-wheel experiment we partially replicated the Lille experiment [3], including elements from the Lyon experiment [6], and added two new conditions (Verbalised reason group and Selective learning group) to test additional systems for knowledge transfer between the artificial generations. Participants were presented with a system that consisted of a four-spoke wheel, the rail on which it travelled at an incline and a time-measurement system (Fig 1, S1 File), according to specifications obtained from Derex et al. [3] and Osiurak et al. [6]. Each wheel spoke had an adjustable weight that could be moved closer to or further from the hub of the wheel. Three conditions were run, each constituting six separated generational transfer chains. Participants were assigned to be part of one of the six chains, where each participant represented a discrete generation in the transfer chain (Fig 2).

Based on the arguments provided by Osiurak et al. [6], participant chains were not sex-segregated, contrary to Derex et al. [3]. In line with the set up for the Lille experiment [3], participants were instructed to perform three tasks in three phases (Table 2):

- in the building phase, minimise the time the wheel took to roll one metre down the track by changing the weight configuration via a computer interface;
- in the testing phase, complete a subsequent knowledge test on weight configurations of the wheel;
- in the theory presentation phase, write a theory for transmission to be read by the next generation.

**Table 2:** Experimental design.

**Building phase**	The building phase started with participants being introduced to the wheel system with 12 discrete positions on each spoke, with all weights in position six. From that they were free to choose their own configurations by using a computer interface. In the interface, each spoke had its own slider with a numerical count of the 12 positions. Once the participant confirmed their configuration, the experimenter positioned the weights accordingly on the physical wheel, all visible to the participant. During the weight placement, the wheel was interlocked in its highest position on the track and held static by a lever. Once the weights were positioned, the participant was notified, and the wheel was manually released by the experimenter using the lever. The time it took the wheel to descend the marked one metre track was automatically recorded by a computer program connected to the interface. The time and the associated configuration were displayed to participants in the same interface used for adjusting the weights. Contrary to Derex et al. [3] and Osiurak et al. [6], the participants viewed their performance in terms of time (ms), rather than in terms of speed (m h^−1^). Each participant had five trials to attempt minimising the time it took the wheel to cover one metre on the inclined track. Participants could at any time consult their latest two configurations, along with the two configurations transferred to them from the former participant. No time constraints were given, and participants could freely roam the interface at their own pace while they choose their next configuration. Participants received information regarding the transfers before starting the building phase. This information included what they would inherit and what they would later pass on to the next participant in the chain. In accordance with Osiurak et al. [6], contra Derex et al. [3], the building phase contained no money incentive. Note, in accordance with the experimental design set up by Derex et al. [3], participants only interacted with the computer interface, never with the physical wheel.
**Knowledge test**	The test was done in a separate computer interface, and participants no longer had access to their results from the building phase. They received instructions stating that they would be presented with 10 separate wheel pairs, and that their task was to assess which of the wheels in each pair would produce the fastest time in the experiment they had just done (Fig 2C, S1 File). The weight configurations of the wheels in this test were directly replicated from Derex et al. [3], with the illustrations adjusted to fit our visual design. All pairs of wheels, as in every line on the test, had to be answered. If a participant was unsure, they were prompted to guess. The option to answer “No difference” from Derex et al. [3], as in both wheels are equally fast, was removed in accordance with Osiurak et al. [6]. Participants had no time constraints and could take as much time as they needed for each pair of wheels. Once a question was answered, the next pair of wheels appeared until all ten questions were completed. As in Derex et al. [3], five of the wheel pairs varied in their moment of inertia. The other five varied in their centre of mass. Participants were not notified of correct or incorrect answers and all participants were introduced to the wheel pairs in the same order.
**Theory for transmission**	After completion of the knowledge test, participants were asked to write down a short theory regarding placements of the weights in the building phase. They received instructions that their theory would be transferred to the next participant, along with their transferred configurations. In accordance with Derex et al. [3] the theories had to be less than 340 characters long and always started with “The wheel covers the distance faster when. . .” (Fig 2B).

From this, we measured three dependent variables: wheel speed, knowledge-test score, and theory-quality score. Knowledge-test score refers to the number of correct answers displayed by participants on the knowledge test which followed the building phase. Theory-quality scores were quantified subsequently to the collection of data. Participants’ suggestions of theory, transmitted to the adjacent participant, were scored independently with regards to the quality of information given based on the two affective dimensions of the wheel (moment of inertia and centre of mass), in the range of 0–2 for each dimension (0 = no correct information; 1 = partially correct information; 2 = full disclosure of correct information). The separate scores of the two dimensions were then combined to a total score ranging from 0–4 (see S1 File). Our condition one (Control group) was a replication of the experimental group detailed in Derex et al. [3] and followed the same set of rules of transfer and theory presentation. This was done to confirm reproducibility of the Lille experimental results [3].

Participants of condition one inherited the last two configurations from the participant before them in the chain of five and were all informed about the nature of the transfer. The configurations and the written theory of transmission from the previous generation, were displayed on the interface as the participant entered the experimental condition. The configurations were explained to be the former participants’ last two trials with corresponding wheel time. Condition two (Verbalised reason group) differed from condition one (Control group) only in the sense that two questions of reasoning were asked by the experimenter during the building phase. The first question: “*What was your process of reasoning when you placed the weights in this way*?*”*, was asked after participant’s second trial. The question: “*How do you think the placement of the weights affects the movement of the wheel*?*”*, was asked after the participant’s fourth trial. The rationale for adding this condition was to test if talking about what you do, enhances understanding [26, 27].

The third condition (Selective learning group) differed from the other two in the means of transfer of trials. While participants in the first (Control group) and second (Verbalised reason group) conditions both received and transferred the last two configurations of each participant, participants in the third condition (Selective learning group) transferred their two best configurations and their corresponding times. The rational for adding the third condition was to test if selective knowledge transfer affects understanding, as humans normally learn from the best teacher [28, 29]. All participants, regardless of condition, had full disclosure of which of their trials would be transferred, and of the nature of their inherited transfer. Information of the other condition instructions were not disclosed to any participant. Participants did not interact with each other.

#### Participants

Written informed consent was obtained from all participants in the study. Ninety participants (47 men and 43 women) took part, varying in age from 19 to 47 (M = 26 years, SD = 5,7 years). All participants were from the University of Gothenburg (students n = 84, faculty staff n = 6), recruited through communication channels within the university (recruitment period: 1 February to 30 April 2022). Continuously testing our results against the Lille [3] and the Lyon [6] experiments, we stopped at 90 participants. By then, adding participants did not change results compared to results from previous versions of the experiment. The language used during the experiment was Swedish, which all participants had full understanding of. All participants completed the experiment. Participants received a movie ticket for completing the experiment.

#### Experimental procedure

All experiments took place in an office located at the University of Gothenburg. Each experiment (20–35 minutes long) followed a similar procedure, with slight differences depending on which of the three conditions the participants were assigned to. All participants were assigned a condition according to the ABBA-principle, e.g., continuously reversing order of the treatment groups. Participants were seated one meter from and facing the physical wheel system (Fig 1). They sat at a table in front of a laptop computer where the interface was accessed (Fig 2B).

#### Data analysis and statistics

We applied a statistical analysis approach similar to Derex et al. [3] and Osiurak et al. [6], where regression was performed with Bayesian statistics (see S1 File for details). A total of five statistical models were used. The first, relating to wheel speed, was modelled by trial number while accounting for the random effects for each chain. Trials where the wheel did not move, and failed to register a speed, were removed from the analysis. The best fitting model by comparing the Bayes Factor between models was the model with trial number with chain as a random effect. Additional models are included in the supplementary material (see S1 File). The second model, relating to the theory-quality score, was modelled by the generation number, accounting for the random effects for each chain, where only the first four trials for each chain were used. For the third, fourth and fifth models, knowledge-test score, theory-quality score, and wheel speed are modelled against each other. All analyses were performed using the R, with the packages *rstanarm* and *bayestestR*. 95% credible intervals (CI) are reported, and Bayes Factor used for evaluating evidence strength and inference. For interpreting evidence strength for and against the null hypothesis using Bayes Factor, we followed the guidelines in Andraszewicz et al. [30]. For all models there were four chains and 40000 samples. All priors were weakly informative by using rstanarm’s defaults.

## Results

**Fig 3:**
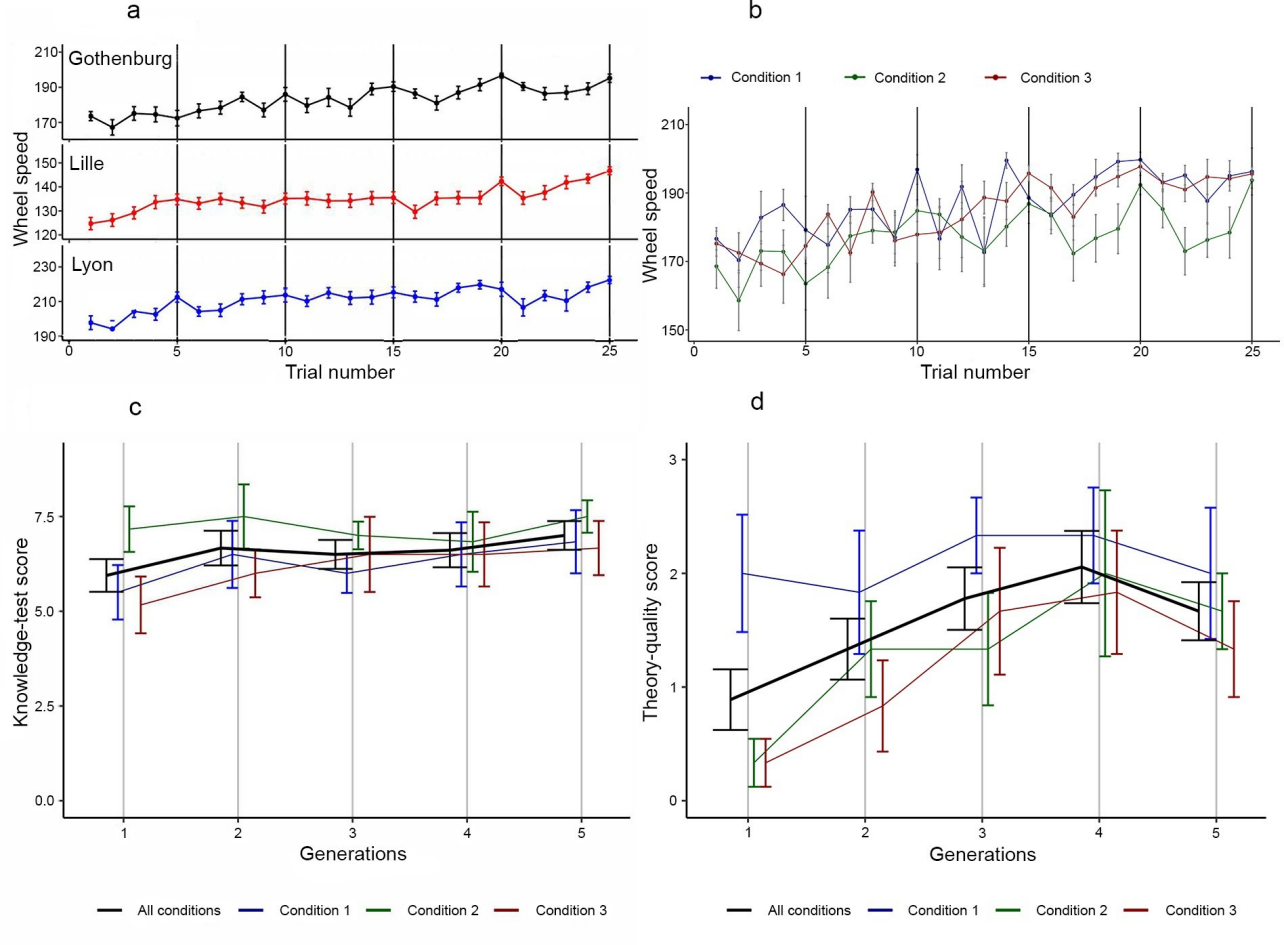
Participants produce faster wheels across generations, their understanding of the system also increases, but peaks at generation four. (**a**) Wheel speed, comparison of the Gothenburg (all conditions), Lille (all conditions) and Lyon (all conditions experiment 1). Maximum speed: Gothenburg = 210 m/h; Lille = 154 m/h; Lyon = 230 m/h. (**b**) Gothenburg wheel speed, separated into conditions: Condition 1 = control group; Condition 2 = verbalised reasoning group; Condition 3 = selective learning group. (**c**) Gothenburg knowledge-test scores. (**d**) Gothenburg theory-quality scores. Error bars show standard deviation.

We were able to confirm reproducibility of the Lille experimental results. The result from the Gothenburg experiment follows the same general trends as those from Lille [3] and Lyon [6]. With generation change, there is a minor speed decline in most of the first trials in all three experiments (Fig 3A). Cumulatively, this indicates that participants initially omitted experiences from the previous generation. Instead, the first trial seems to reflect self-exploration through trial and error. Similar trends in all the experiments demonstrate that, regardless of experimental conditions, generation four always came close to obtaining the theoretical maximum speed of the abstracted-wheel system (Fig 3A). Due to participants having no prior knowledge about the limit value of a theoretical maximum speed, the only option for participants in generation five was to first diminish their performance when shifting the weights, before also reaching the theoretical maximum (Fig 3A).

We see a general progression of wheel speed in all three Gothenburg conditions (Fig 3B, trial number: posterior median: 0.87 m/h-1, 95% CI = [0.68, 1.06] BF>100, extreme evidence, see S1 File), and the combined results over generations of the knowledge test show a slight increase (Fig 3C). Between conditions, the results are comparatively scattered in the first three generations, converging in generations four and five indicating that our added conditions did not affect results compared to previous experiments [3, 6]. Participants were able to form ideas of the underlying principles of the functionality of the wheel, with the first four participant generations of the Gothenburg experiment showing an improved ability to formulate a theory (Fig 3D). Theory-quality scores increase significantly from generation one through four (Fig 3D, median = 0.39, 95% CI = [0.18, 0.61], BF = 17.22, strong evidence). Reading a theory transferred from previous generation, seems to improve participants’ understanding of the wheel system. As the participants in the fifth generation of the Gothenburg experiment knew that they were the last generation, and also knew that their theory descriptions would not be used by future generations, the decline for this generation is likely attributed to a lack of motivation (Fig 3D).

**Fig 4:**
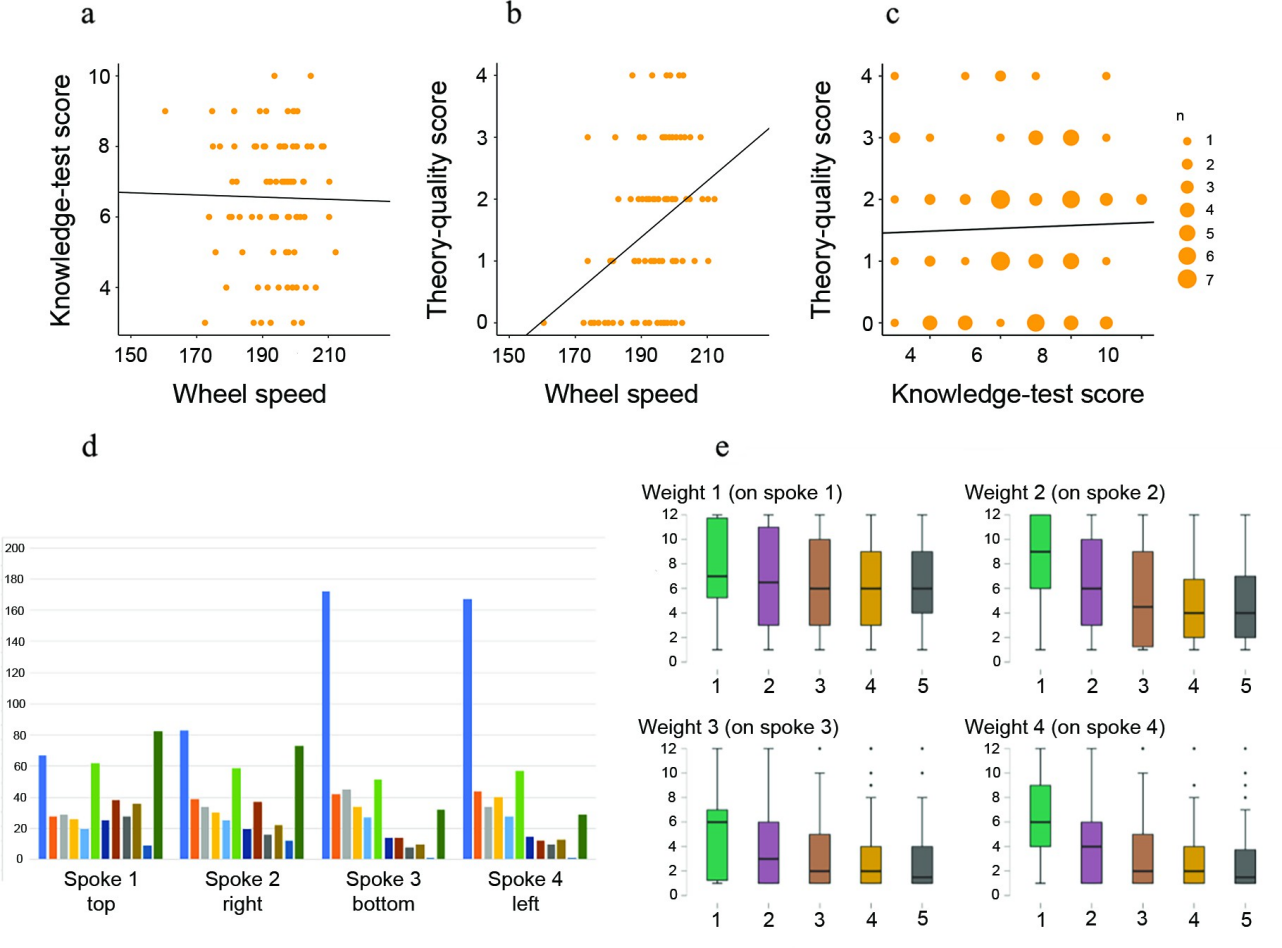
In the Gothenburg experiment, participants increased their reasoning ability, and ability to convey useful theories by going through the ‘building phase’. Participants followed what is understood as a reasonable set of configurations in weight placement. They optimised the wheel by an inter-generational process of eliminating less-fit alternatives. (a) Correlation between knowledge-test scores and wheel speed, showing moderate evidence for null hypothesis (wheel speed: posterior median: -0.003, 95% CI: [-0.04, 0.04], BF = 0.043). (b) Correlation between theory-quality score and wheel speed, showing strong evidence for the correlation (wheel speed: posterior median: 0.05, 95% CI: [0.02, 0.07], BF = 26.35). (c) Correlation between theory-quality score and knowledge-test score, showing moderate evidence for the null hypothesis (knowledge-test score: posterior median: 0.02, 95% CI [-0.12, 0.17], BF = 0.045). (d) Bar graphs of the number of weight placements on each spoke for position 1 (blue bar to the left) to position 12 (green bar to the right). (e) Box plot of weight placement conformity (position 1–12) over generations (1–5).

The Gothenburg results show a lack of correlation between the knowledge test that is set up to compare and eliminate between options and the optimisation of wheel speed (Fig 4A). On the other hand, there is strong evidence of a relationship between the theory-quality score and the optimisation of wheel speed (Fig 4B). Thus, participants going through the building phase increased their reasoning ability and their ability to convey cohesive and correct theories about the technical system, in tandem with their ability to produce faster wheels. These observations demonstrate an understanding of both the task performed and the causality of the wheel system. The lack of correlation between the knowledge-test scores and wheel speed (Fig 4A), in combination with strong evidence that there is no correlation between the knowledge-test scores and the theory-quality scores (Fig 4C), implies that the knowledge test does not assess improved understanding related to wheel-building. Indeed, the abstracted-wheel experiment is set up for participants to train in *building* wheels, but their knowledge is tested by their ability to *analyse* and *compare* differences between wheels. From this we conclude that what is trained for is not what is tested for in the knowledge-test. The original experimental set-up was therefore flawed in this respect.

We see a trend in that participants follow what is understood as a reasonable set of configurations in weight placement, indicating a pre-experimental understanding of the weight system (Fig 4D). We speculate this also to be partially based on cueing, as an effect from the initial weight placements when a participant is first introduced to the system (see Figs 1 and 2A). Weight placement conformity (Fig 4E) shows that the abstracted-wheel experiment is not about improving a technical artefact. Instead, it is about the ability to differentiate between reasonable and unreasonable weight placements through an inter-generational process of eliminating the number of less-fit alternatives to reach a best-fit solution.

## Discussion

**Fig 5:**
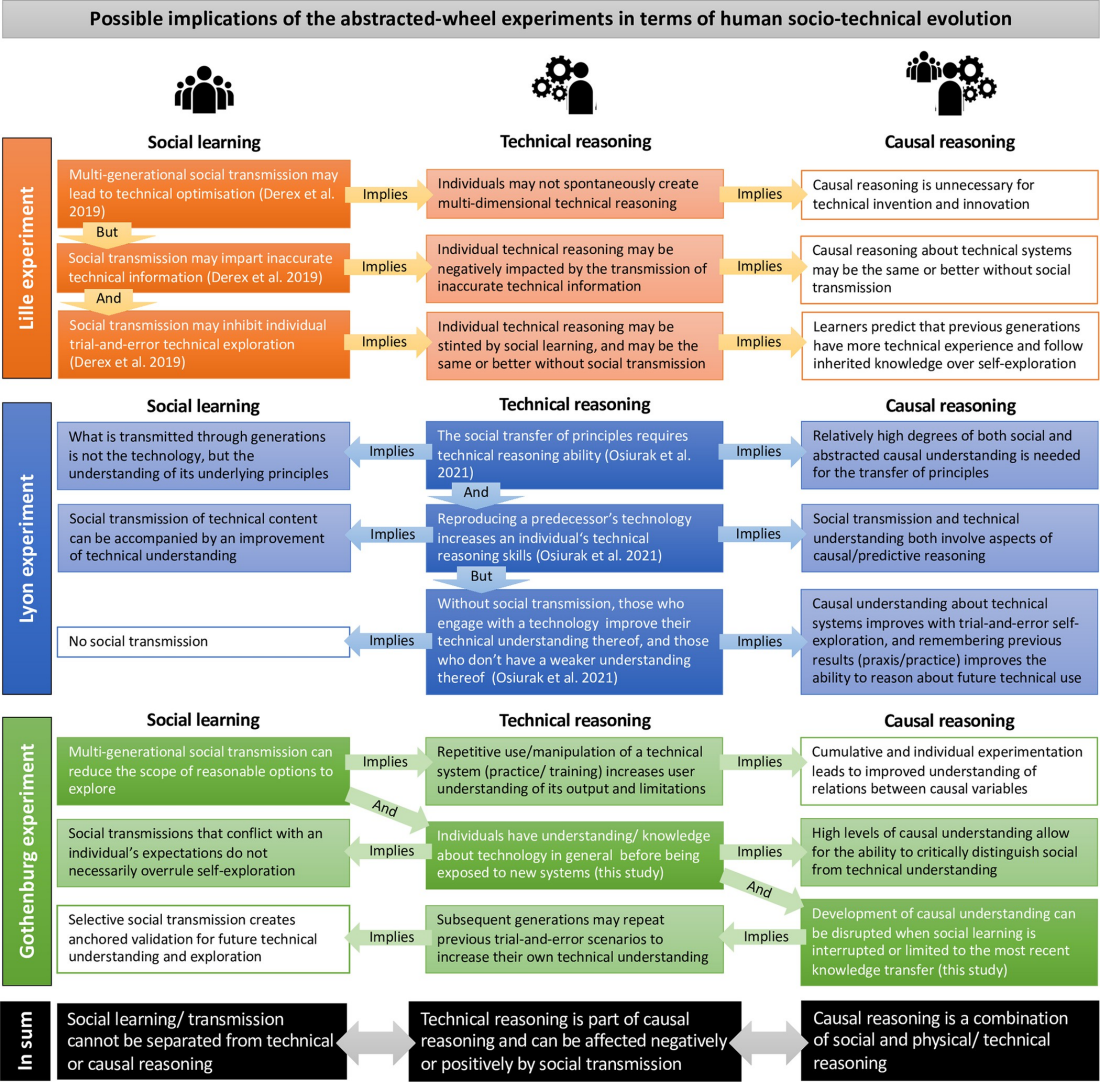
What the micro-society abstracted-wheel experiments may reveal in terms of socio-technical evolution. **Orange blocks**: The Lille experiment [3] with aspects of their outcomes in the dark blocks with white text, and our reading of the potential implications for technical and causal reasoning in the lighter boxes with black text. **Blue blocks**: The Lyon experiment [6] with aspects of their outcomes in the dark blocks with white text, and our reading of the potential implications for social transmission and causal reasoning in the lighter boxes with black text. **Green blocks**: The Gothenburg experiment with aspects of our outcomes in the dark blocks with white text, and our reading of the potential implications for social transmission, technical and causal reasoning in the lighter boxes with black text. **Black blocks**: Our conclusions.

To assess what the abstracted-wheel experiments may or may not reveal about human socio-technical evolution, we identified key outcomes, or what we interpreted as potentially important evolutionary aspects from each of the abstracted-wheel experiments and the theoretical account they speak to (Fig 5 dark blocks with white text). We then used published results from the Lille [3] and Lyon [6] experiments, together with results from the Gothenburg experiment presented here, to assess what each of these may imply regarding the theoretical accounts of social learning, technical reasoning and causal reasoning elaborated on in the different versions of the abstracted-wheel experiment (Fig 5 horizontal arrows and lighter text boxes).

A key outcome of the Lille experiment [3] was that multi-generational social transmission would lead to technical optimisation (Fig 5 first orange block with white text). This would imply that individuals may not spontaneously create multidimensional technical reasoning, and that causal reasoning is unnecessary for technical invention and innovation. However, Derex et al. [3] also show that social transmission may impart inaccurate technical information (Fig 5 second orange block with white text), which in an evolutionary context would imply that individual technical reasoning may be negatively impacted. Thus, for technological cumulation and invention as observed throughout the Pleistocene record, causal reasoning about technical systems may at times have been the same or better without social transmission. The same would imply for technical reasoning when Derex et al. [3] discuss how social transmission may inhibit individual trial-and-error technical exploration (Fig 5 third orange block with white text) and, thereby stint self-exploration and having “negative downstream effects on participants’ performance”. This is in line with Stuart-Fox [31] who notes that ideas about causal connections that an individual can form are prone to errors. Consequently, if the next generation predicts that the previous generation has more technical experience, and prefer to follow inherited knowledge over self-exploration, it implies theory of mind or social causal reasoning. Thus, even though the Lille experiment set out to show that evolving technology and causal reasoning are disconnected, its outcomes do have possible evolutionary implications in terms of both technical and causal reasoning (Fig 5 orange blocks).

The key argument of the Lyon [6] abstracted-wheel experiment was that technical reasoning is important for cumulative technological culture, and that the social transfer of technical principles requires technical reasoning ability (Fig 5 first blue block with white text). This could imply that in some instances during the Pleistocene, it was not a technology or technique that was transmitted through the generations, but the understanding of some of its underlaying principles, in which case relatively high degrees of both social and abstracted technical causal understanding would be in play. This is in line with Reed et al. [32] who discuss social learning as something that goes beyond the individual to become situated within a wider socio-technical practice. The Lyon experiment [6] also showed that by reproducing a predecessor’s technology, the technical reasoning skills of an individual can be increased (Fig 5 second blue block with white text), which implies that the social transmission of technical content may be accompanied by an improvement of technical understanding. Causal or predictive reasoning, as discussed by Gärdenfors and Högberg [33], is involved in both these processes.

Importantly, Osiurak et al. [6] also demonstrate that, without any social transmission, people who engage with a technology would improve their technical understanding thereof, whereas those who do not actively engage have a weaker understanding of the technical principles (Fig 5 third blue block with white text). In the Gothenburg experiment for example, some participants initially omitted experiences from the previous generation to instead engage with self-exploration through trial and error. Along similar lines as Donald [34], we argue that this observation underpins the notion that causal understanding about technical systems improves through trial-and-error self-exploration. For instance, by remembering past technical performances and subsequent physical rehearsal (practice) and/or mental rehearsal (praxis) future technical performance can be improved [34, 35] (Fig 5 blue blocks).

The Gothenburg experiment set out not to argue for, nor against, any of the outcomes from previous versions of the abstracted-wheel experiment. Instead, we aimed to assess the potential of the abstracted-wheel experiment for informing aspects of the evolution of human technical behaviour. In the process, we found that such behaviour cross-cut aspects of social learning, technical reasoning, and causal reasoning (Fig 5 green blocks and diagonal arrows). In terms of the experiment, we show that multi-generational social transmission reduces the scope of reasonable options to explore through a process of elimination. This may imply that repetitive (generational and individual) use or manipulation of a technical system would also increase a user’s understanding of its potential output and limitations. Combined, such cumulative and individual experimentation during the Pleistocene may have led to an improved understanding of relations between causal variables within technical systems. This is in line with previous theoretical discussions on human socio-technical evolution that elaborate on a holistic approach to understand social learning, technical reasoning, and causal reasoning [34].

Even though Derex et al. [3] designed the experiment to represent an unfamiliar technology, we found that individuals come with a certain level of general technical knowledge and understanding that they transfer to the new system. This is exemplified in the writing of theory transmissions. For example, one participant in the Gothenburg experiment wrote that ‘The wheel covers the distance faster when it gains momentum early on and the weight doing this is primarily applied to one spoke’. Also, transmitted knowledge about the abstracted wheel that conflicted with generalised self-knowledge, did not always overrule self-exploration. We also found that when social learning is interrupted or limited to the most recent transmission, the development of causal understanding too can be disrupted. For example, by knowing that they were the last generation in the series, our last groups lacked the motivation of previous generations to develop useful theories for social transmission. Hence, the result of their technological engagement appeared in “accordance with social strategies” ([23], page 5). This would imply that future generations or different groups who re-invent a technology would have to repeat previous trial-and-error scenarios to increase their own technical and causal understanding thereof. The selective social transmission of technical solutions (i.e., keeping the solutions in the communal mind) thus creates an anchored validation from which the next generation can evolve their understanding and explorations (Fig 5 green blocks).

In sum, the three abstracted-wheel experiments (Lille, Lyon and Gothenburg) altogether may reveal some aspects about human socio-technical evolution throughout the Pleistocene. The inferences are however not as cut-and-dry as Derex et al. [3] presented them, because our analysis shows that (Fig 5 black blocks):

1. Social learning cannot be separated from either technical or causal reasoning.
2. Technical reasoning can be affected either negatively or positively by social transmission and is inherently part of causal reasoning.
3. Causal reasoning is a combination of both social and technical reasoning.

**Fig 6:**
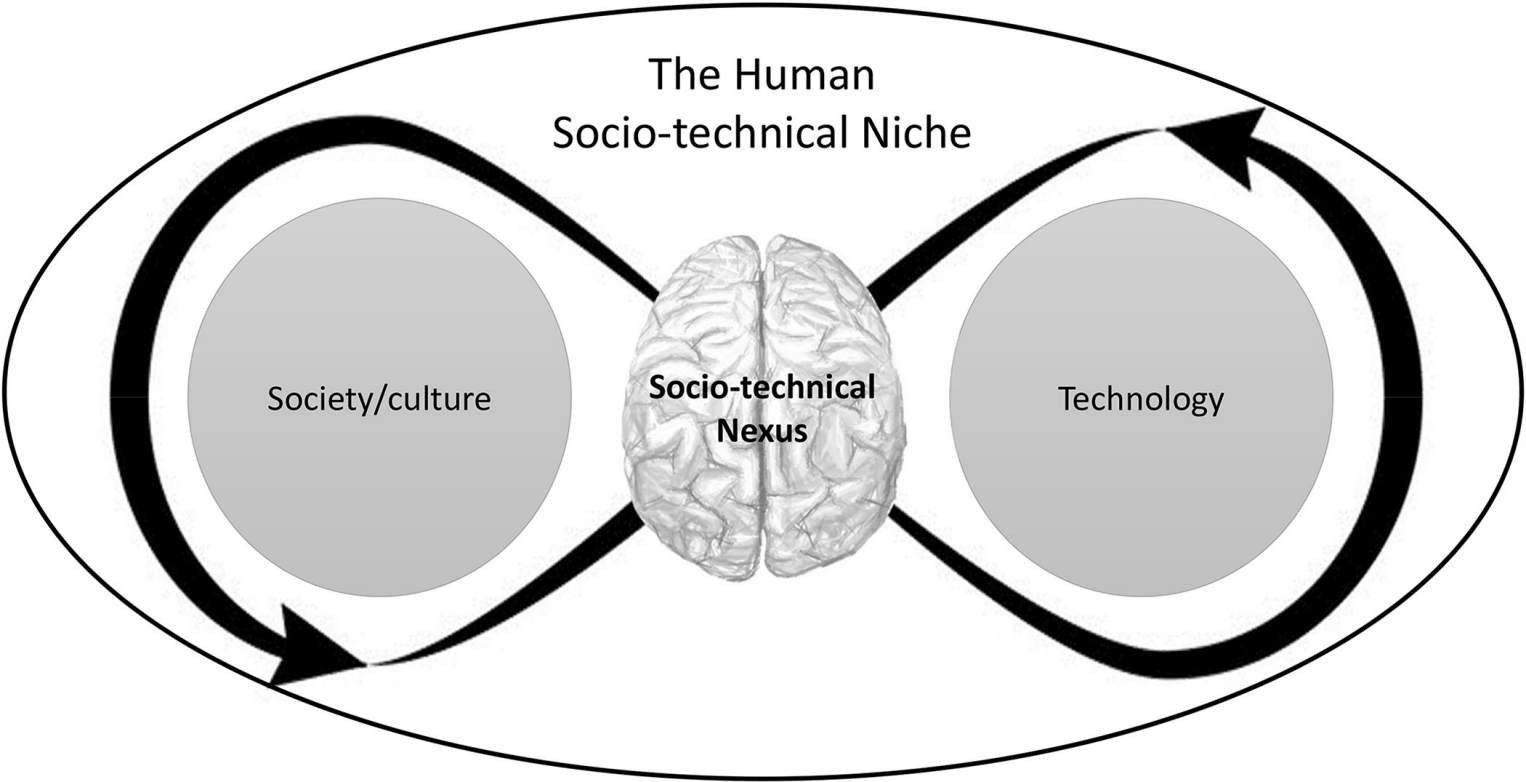
A co-evolutionary position. Human cognition (individual and collective extended mind [38, 39]) serves as the nexus for processing information from both the social and the technical domains, stimulating the innovation and use of increasingly complex technologies.

From this, we suggest that a co-evolutionary position–interweaving various aspects of social learning, technical reasoning, and causal reasoning–that offers a robust explanation for the human socio-technical niche (Fig 6, see also [8–11, 21, 36, 37]).

### Our results in a wider context

Before drawing conclusions about what the abstracted-wheel experiment may reveal about human socio-technical evolution, it is necessary to take a closer look at some of the confounding aspects of micro-society experiments in relation to evolutionary studies (Table 3). Mesoudi ([12], C6P31) argues that even though “experiments should be viewed as tests of theories rather than exact simulations of the real world, there should nevertheless be some kind of link to [the] real-world […] for an experiment to be useful”. Along these lines, a particularly confounding feature of the abstracted-wheel experiment is the fact that participants used computer interfaces with abstracted representations of a technology, instead of manually manipulating the technology itself. Their bodies and minds thus remain detached from visuo-haptic and social engagement relating to the physical wheel–i.e., they are separated from the situated cognition inherent in technical/material engagement with the technical artifact [40]. Indeed, participants in our experiment at first often did not even notice the physical wheel system in front of them. Instead, they were surprised to discover it as a real-world representation of their virtual-wheel manipulation. As discussed by Mesoudi ([12], C6S1), this is a problem not unique to the abstracted-wheel experiment, but also common in other forms of micro-society laboratory experimental studies that are “constrained within artificial boundaries”.

**Table 3:** Challenges associated with using a micro-society laboratory experimental study as the abstracted-wheel experiment as proxy for human socio-technical evolution throughout the Pleistocene.

Aspect	Ambiguous real-world settings	Controlled laboratory conditions
**Previous criticism** (modified from Miton and Charbonneau [56]; Caldwell et al. [57]; O´Brien and Bentley [58]; Packer and Cole [51]; Scott-Phillips [59]; Nichols et al. [60])		
**Environment**	Large-scale settings in various ecological and social environments	Strictly limited artificial environments
**Time**	Several biological generations, millennia of shared ancestral history	Artificial generations wherein processes are limited by the time spent with the experiment
**Temporality**	Reasons for performing and acting are set in a *longue durée*, interrelated with long-term historical structures and future anticipation	Acting is a-historic, with a non-existing before and after
**Population/demography**	Large-scale spatiotemporal and cultural variation	Limited or no variation, mostly from WEIRD societies
**Technology**	Rich spatiotemporal variation, technology is used as a means of surviving and not for the sake of the technology in itself	Modern technology with no, or strictly limited, variation, used as an outcome in itself. Not related to survival
**Our observations**		
**Motivation**	Technologies and techniques are goal-directed steps towards acquisition and/or avoidance wherein multidimensional planning is fundamental	Motivation relies solely on task completion, sometimes associated with a reward unrelated to the task, planning is rarely possible outside of the given situation
**Embodied cognition**	Touching and observing three-dimensional objects in real-life space-time provides information about the physical traits of a technical system and how it works–it shapes neural pathways for effective technical engagement	Engagement through a replicated computer interface is inherently different from manually engaging with the represented real-life technology in a real-world setting, resulting in a visuo-haptic disconnect cognitively–it engages neural pathways in relation to interacting with the interface, not with the three dimensional abstracted-wheel
**Prior knowledge**	Humans do not operate in technical vacuums, object-play from a young age and throughout life explores physical/technical traits of objects and how they function	The abstracted wheel was originally used because it was deemed unfamiliar to WEIRD students [3], thus unnaturally devoid of prior knowledge
**Literacy**	Most of human socio-technical evolution was devoid of literacy, today people still learn to make or use technologies without being literate	Literacy is a key element in most micro-society studies
**Quantitative *vs* qualitative**	Human socio-technical behaviour and the evolution thereof likely represent qualitative variation and change in behaviours, difficult or impossible to quantify	Micro-society experimentation leans heavily on quantification instead of qualitative variation
**Invention/innovation**	Confronted with a new problem, some people will be inspired to invent novel solutions while others will adapt existing ones	If the experiment does not include the possibility of invention and innovation, it cannot test for invention or innovation

Physically interacting with a technology while being in both natural and socio-technical environments, affects a person’s perception, learning, reasoning, decision making, and how they act and react [40]. Over the past two or three million years, interaction with technologies in varying ecological and social settings shaped aspects of our bodies, brains, and minds [10, 33, 41–46]. Both the first and subsequent generational experiences with the abstracted-wheel system are in stark contrast with the life-long, embodied learning curves and causal understanding associated with hunter-gatherer techno-behaviours, such as bow hunting [11, 35, 45–49].

The abstracted-wheel experiments necessitate interacting with information that requires literacy, a mouse, screen, and touchpad/keyboard in a limited, controlled, sedentary and largely de-socialised setting. This is intrinsically different cognitively (socially, technically, and causally) from the continuous learning and teaching processes involved in Pleistocene techno-behaviours, such as collecting the materials to construct your own bow and poisoned arrows, tracking game, shooting it, butchering it, and transporting it with the purpose to provide food for your group’s survival [45–47]. Along these lines, we speculate that the abstracted-wheel experiments probably reveal more about WEIRD (western, educated, industrialised, rich, democratic) populations [50–53], than about embodied socio-technical evolution throughout our pre-literate *Homo* lineage [10, 33, 41, 42, 44, 54, 55]. Also, through their Western formal education participants in all three experiments were all familiar with basic laws of physics. This is evident in that they, although facing an abstracted wheel that is intuitively difficult to understand (as was the intention in the first Lille experiment), they were able to form ideas about the principles underlying the function of the wheel. As suggested by Derex et al. ([3], page 449), work involving what they call “non-WEIRD participants” may further explore aspects of what is culturally constructed or shared across populations in terms of results from the abstracted-wheel experiment. Another way forward would be to design protocols wherein participants are able to manually manipulate the technology, and to design tests that can explicitly address aspects of social learning, technical reasoning and causal reasoning in such contexts.

The original Lille experiment ([3], page 446) tried to explore social learning “in the absence of explicit causal understanding”. The next generation of the experiment, the Lyon experiment [6], demonstrated the opposite by showing that technical reasoning (an aspect of causal reasoning) is indeed important for cumulative technological culture. We argue that it is not possible to extract causal reasoning about technology from the *Homo sapiens* social context. These domains almost certainly co-evolved throughout the Pleistocene to become seamlessly integrated into our humanness today [40, 61, 62]. We see that the abstracted-wheel experiment, instead of testing cognition, tend to reflect its behavioural output only. The ability of such tests to inform on aspects of Pleistocene cognitive evolution is therefore limited.

We conclude that the reduction of variables tested with the abstracted-wheel experiments in controlled, micro-society, time-constrained, asocial settings cannot analyse causal understanding about the optimisation of a multidimensional technology, nor can it assess the role of cultural/social transmission in the evolution of human techno-behaviours. Therefore, it cannot model or serve as robust proxy for the evolution of Pleistocene socio-technical complexity expressed in the invention, use and improvement of the bow-and-arrow, architecture, or watercraft, as claimed by Derex et al. [3]. What the abstracted-wheel experiments (Lille, Lyon, Gothenburg) have done, is to give us valuable insights into the interrelatedness of social learning, technical reasoning, and causal reasoning, that in turn, may help us to reveal aspects about human socio-technical evolution throughout the Pleistocene.

## Supporting information

S1 File Supplementary material for the Gothenburg experiment. (DOCX)
