# Supplementary material for: Human socio-technical evolution through the lens of an abstracted-wheel experiment: A critical look at a micro-society laboratory study
Source: PLoS One. 2024 Nov 11;19(11):e0310503. doi: 10.1371/journal.pone.0310503 (PMC11554191; doi:10.1371/journal.pone.0310503)
Supplement: S1 File — (DOCX) [file pone.0310503.s001.docx]

**S1 File. Supplementary material for the Gothenburg experiment**

**Ethical statement**

The study was carried out in accordance with the ethical standard of the Swedish Research Council. Participation was voluntary and all participants provided written, informed consent before taking part in the experiment. Participants could withdraw their consent at any time. Data was handled confidential. Participant were anonymised. The study is of a sort that do not require ethical review in accordance with the requirements of the Swedish Ethical Review Act, 2003:460, paragraphs 3 and 4, that concerns the ethical review of research involving humans.

**Experimental apparatus**

*Building of the wheel*

The wheel with its four spokes and the centre axis were held together with a three-axle tube clamp. The spokes as well as the axis were made from a wooden round-bar with 28 mm in diameter. The spokes extended 410 mm from the centre of the axis and were marked up in 28 mm 12 discrete segments with red heat shrink tubing. The centre axis fitted perpendicular to the spokes through the three-axle tube clamp, extended differently on either side of the tube clamp to compensate for the asymmetric design of the tube clamp. The length of the centre axis wooden bar was the sum of the distance through two 500 g weight discs, two barbell clamps, one 11 mm spacer and one three axle tube clamp. An 8 mm longitudinal hole through the centre axis was machined on lathe to obtain a centred straight hole. An 11 mm thick plywood spacer was cut out and machined on lathe and mounted around the centre axis on the side of the tube clamp where the centre axis extended longer. The purpose of the spacer was to keep the 500 gr weight discs on equal distance from the centre of the spokes, which was critical to maintaining a straight path along the rail track. A M8 threaded steel rod was snuggly fitted through the hole in the centre axis and on either side 40 mm rubber tube and two pairs of nuts to lock all parts in place. The purpose of the rubber tube was to prevent sliding against the rails, while the locking nuts had the purpose to prevent the rubber tube from twisting around the threaded steel rod.

Four pieces of adjustable weights were composed out of barbell clamps, multiple metal washers held in place with M5 bolts and nylock nuts. Each unit weighed 100 g ± 0.5 g, controlled on a two decimal digital scale, and placed along each spoke. The weight units were composed of the same type of barbell clamp that were used to lock the 500 g weight disc on the centre accel in position.

To battle a wider than tolerable dimensional range in the accuracies of many of the individual parts, the wheel was fixated in a jig to maintain perpendicularity of the spokes and centre accel. The spokes and accel were glued together inside the three-axle clamp with expanding polyurethane glue. The specific glue was picked for its ability to glue metal together with wood as well as for filling out the excess space in the tube clamp making a gapless bond able to withstand the forces involved in the experiment. One design problem found during the study was that the rubber tube on the centre axis got unevenly squeezed lengthwise, thereby radially expanded, causing a difference in radius on either side and imposing a slight deviation from the wheel spinning straight on the rails. A similar twisting problem occurred between the weight discs and the wooden centre axis. During the experiment the problematic sectional parts mentioned above were continuously replaced to avoid confounding performance of the apparatus.

*Building of the rails*

The rail structure, along which the wheel spun, was welded, and bolted together out of different types of aluminium profiles. The rails were constructed of two separate 2000 mm long aluminium L profiles, positioned in a 14-degree angled slope. Two stop blocks made from triangular shaped low friction plastic were bolted in the lower end of the rails. The aim of the shape was to let the wheel slidingly climb a few centimetres and gradually decrease rotation speed. The frame structure holding the rails were made 40 mm wider than the width of the rails to give space in case of further adjustments for the wheel to spin free of sideways friction from the rails. A counter balanced lever was placed in the highest part of the frame structure right above the start position of the wheel. The lever held the wheel in the desired starting position, perpendicular to a horizontal line, enabling the wheel to maintain an equal starting position for each start. In each four ends of the two base beams 8 mm vertical holes were drilled to hold bolts providing adjustable feet to obtain levelled support for the rail.

*Interface and time measurement*

The design of the interface was copied with modifications from Derex et al (2019). A web interface together with the timing was made up of three separate parts that work together. Firstly, an ESP32 microcontroller was used for the actual timekeeping. The microcontroller ran a code written in Arduino Programming Language and was connected to the start and stop switches on the apparatus’ rail. Two time-switches placed at the start and at the 1 m point of the track forwarded events to the server when the voltage in the switches changed. Secondly, a server written in Node.js handled communication with the ESP32. The server stored data in a csv file from which the web interface could download the data through a REST API. The server also forwarded the time measurements from ESP32 to the interface through web sockets (socket.io). Thirdly, a web interface written in Javascript built with React that handled the regulation and display of data available to participants. The interface retrieved data from the server through a REST API and updated new data through the same API. A web socket connection was established, for each trial through validation, that listened for a triggering event containing new data output.


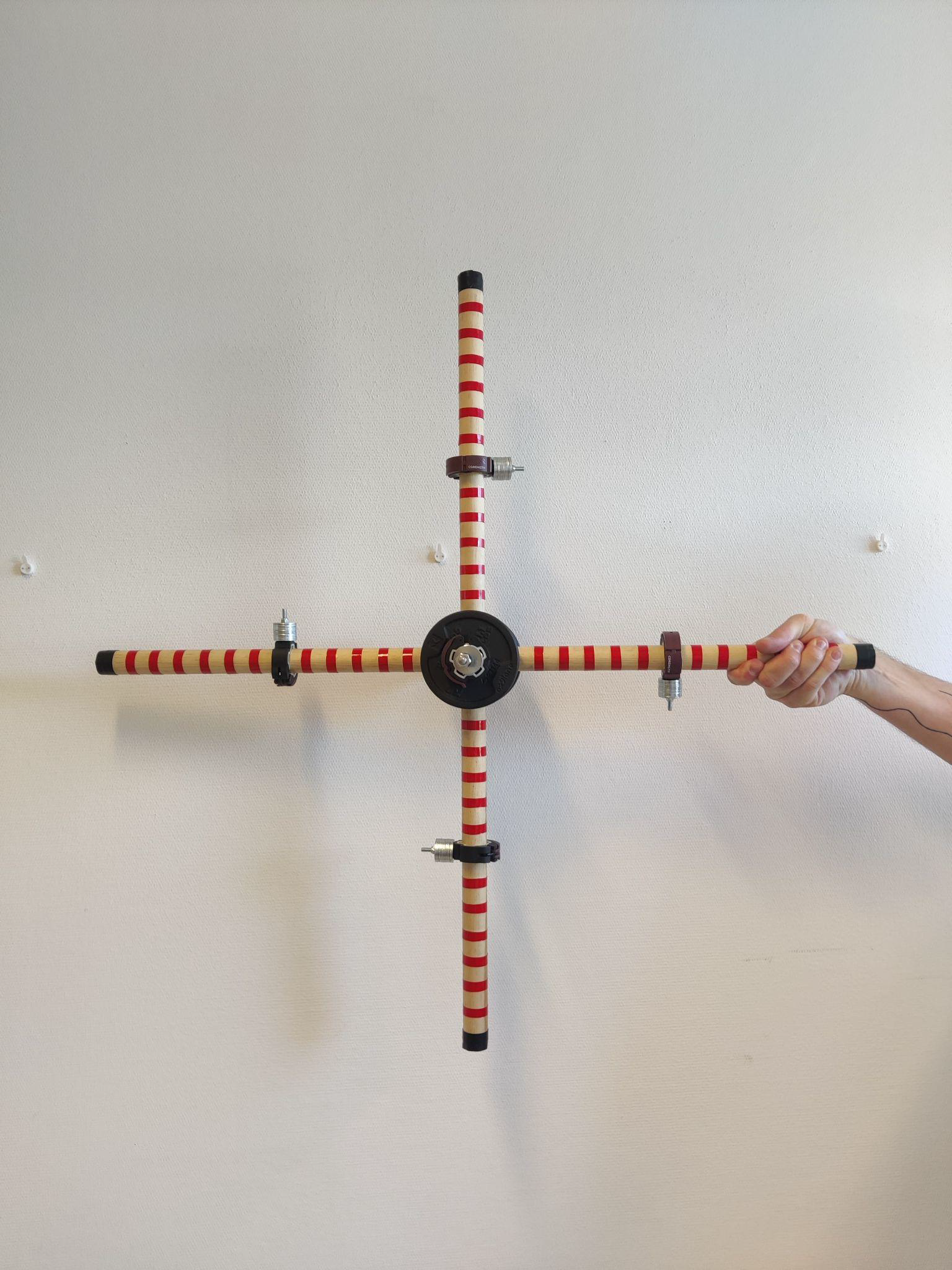


The wheel that was used throughout the study. Each of the red stripes marks a discrete position on which a weight could be placed. The weights here are placed at position six on each of the four spokes, which was also the initial position before each participant was presented with the option of alteration.


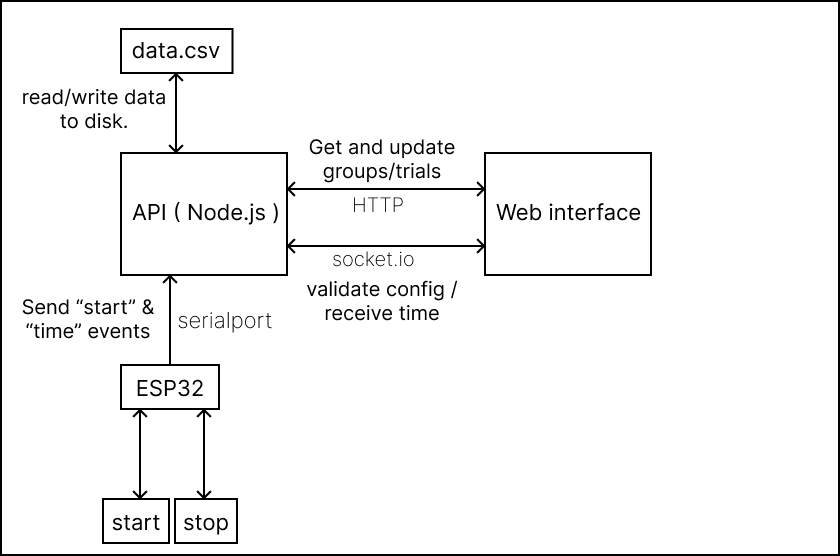


Flow Scheme. Time keeping and interface.


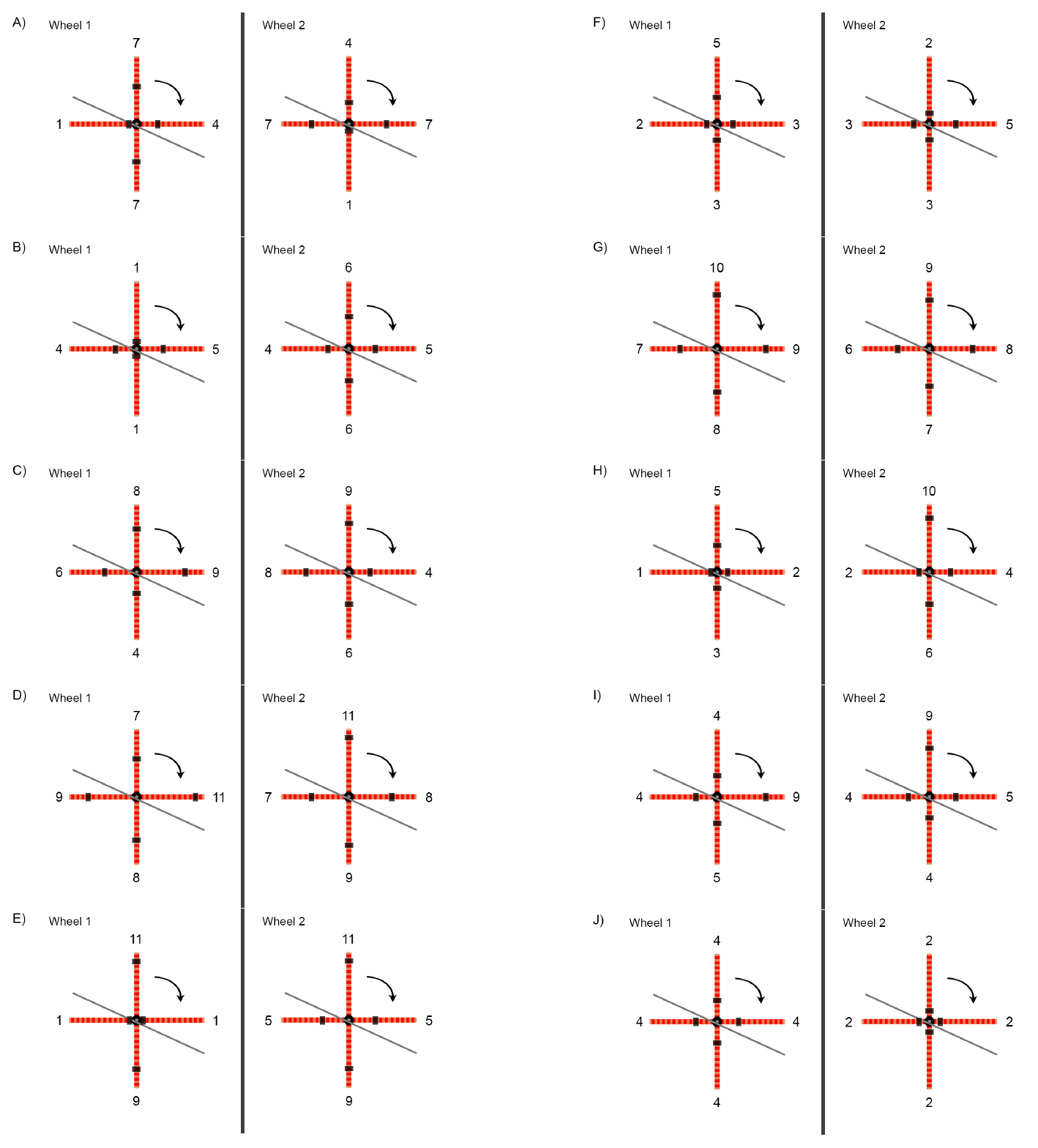


The complete knowledge test, each wheel pair presented separately for participants. Wheel pairs A, C, D, F, and I differed in centre of mass distribution. Wheel pairs B, E, G, H and J differed in their moment of inertia. Correct answers: A (2), B (1), C (1), D (2), E (1), F (1), G (2), H (1), I (2), J (2).

**Knowledge-test score and theory-quality score**

Knowledge-test scores refers to the number of correct answers displayed by participants on the knowledge test which followed the building phase. The scores were collected individually for each participant. Every correct answer rendered one point and the range of possible scores were 0 (zero, minimum) to 10 (ten, maximum). Theory-quality scores were quantified subsequently to the collection of data. Participants’ suggestions of theory, transmitted to the adjacent participant, were scored independently with regards to the quality of information given based on the two affective dimensions of the wheel (moment of inertia and centre of mass). A score was given in the range of 0–2 for each dimension based on these criteria: 0=no correct information; 1=partially correct information; 2=full disclosure of correct information. The separate scores of the two dimensions were then combined to a total score ranging from 0–4. Grading of the theory-quality scores was performed blind to treatment and generation by the experimenters. Experimenters first graded the full data set independently and later in collaboration to map out the final theory-quality score. All dependent variables were measured and collected separately.

**Comparison of Statistical Models for Wheel Speed**

In the model corresponding to Fig 3b, as presented in the main text, wheel speed is modelled by trial number, while accounting for the random effects associated with each chain. Notably, the three different conditions were not included in this particular model. To assess whether the inclusion of the condition variable improves the model's fit, we evaluated the following models using R formula syntax:

- Model-0: Wheel Speed ~ 1 + (1 | Chain)
- Model-1: Wheel Speed ~ 1 + trial number + (1 | Chain)
- Model-2: Wheel Speed ~ 1 + trial number:condition + (1 | Chain)
- Model-3: Wheel Speed ~ 1 + trial number + condition + (1 | Chain)

Here, the "condition" variable is categorical. Model 2 considers an interaction between trial number and condition, while Mode 3 treats "condition" as a fixed effect, both representing different approaches to examine whether the slope of wheel speed varies depending on the condition. In all models, "Chain" is included as a random intercept since each starting value can differ.

These models are subsequently compared to model-0 using Bayes factor (R package: BayesFactor, Bayes Factor type: marginal likelihoods from bridgesampling) to determine which one better fits the data. With Model-0 as the denominator, strong evidence (BF>>100) was found in favour of all three models, with Model-1 showing a higher preference. This implies that trial number should be incorporated into the model.

To explicitly investigate whether either of the two models with "condition" included results in a better fit than Model-1, we placed model-1 in the denominator. Model-2 compared to Model-1 resulted in BF=0.284, while Model-3 compared to model-1 yielded a BF of 0.222. In summary, the best-fitting model did not include the modelling of the condition variable. Therefore, only Model-1 is presented in the main text.

**References**

Derex, M., Bonnefon, J-F., Boyd, R. & Mesoudi, A. 2019. Causal understanding is not necessary for the improvement of culturally evolving technology. *Nature Human Behaviour* 3: 446–452 <https://doi.org/10.1038/s41562-019-0567-9>

Osiurak, F., Lasserre, S., Arbanti, J., Brogniart, J., Bluet, A., Navarro, J. & Reynaud, E. 2021. Technical reasoning is important for cumulative technological culture. *Nature Human Behaviour*, 5: 1643–1651. <https://doi.org/10.1038/s41562-021-01159-9>
